# Supplementary material for: Inhibition of ErbB kinase signalling promotes resolution of neutrophilic inflammation
Source: eLife. 2019 Oct 15;8:e50990. doi: 10.7554/eLife.50990 (PMC6839918; doi:10.7554/eLife.50990)
Supplement: Supplementary file 1. — PKIS compounds were incubated with neutrophils for 6 hr and apoptosis was assessed by Annexin V/TO-PRO-3 staining by flow cytometry. Sixty-two compounds accelerated apoptosis ≥2 fold and compound names are presented here, along with fold change over control. The kinase profiling information for each of these inhibitors is available to download at https://www.nature.com/articles/nbt.3374#supplementary-information (file ‘PKIS Nanosyn Assay Heatmaps’ from Supplementary Data) (Elkins et al., 2016). [file elife-50990-supp1.docx]

| Compounds | Apoptosis Fold change (of DMSO control) | Compounds | Apoptosis Fold change (of DMSO control) |
| --- | --- | --- | --- |
| GW589933X | 8.63 | GW576924A | 2.69 |
| GW305074X | 8.57 | GW297361X | 2.64 |
| GW441756X | 8.52 | GSK317354A | 2.64 |
| GW305178X | 7.26 | GW445017X | 2.60 |
| GSK579289A | 6.37 | GW567808A | 2.56 |
| GSK943949A | 4.65 | GW831091X | 2.53 |
| GSK1751853A | 4.46 | SB-686709-A | 2.47 |
| SB-278538 | 4.21 | GW832467X | 2.44 |
| GW631581B | 4.08 | SB-376719 | 2.41 |
| GW513184X | 4.06 | GSK237700A | 2.40 |
| SB-245392 | 3.99 | GW440139A | 2.39 |
| GW830365A | 3.89 | GW820759X | 2.39 |
| GW795493X | 3.87 | GW406108X | 2.37 |
| SB-751148 | 3.85 | GSK204925A | 2.31 |
| GSK1000163A | 3.79 | GSK994854A | 2.29 |
| GSK978744A | 3.78 | GW827106X | 2.27 |
| GW442130X | 3.71 | GW683109X | 2.27 |
| GW627834A | 3.68 | SB-736290 | 2.25 |
| GSK1326255A | 3.64 | GW814408X | 2.17 |
| SB-242719 | 3.59 | GW829055X | 2.16 |
| GW831090X | 3.56 | GW580496A | 2.11 |
| GW569293E | 3.36 | GSK614526A | 2.10 |
| GW680908A | 3.34 | GW781673X | 2.09 |
| GSK237701A | 3.33 | GW616030X | 2.09 |
| GSK1173862A | 3.23 | GW770249A | 2.08 |
| GW296115X | 3.04 | GW301784X | 2.07 |
| GW574782A | 3.04 | GW694590A | 2.05 |
| GW784307A | 3.04 | GW607049C | 2.04 |
| GW796920X | 2.75 | GW407323A | 2.02 |
| GW284372X | 2.72 | SB-278539 | 2.02 |
| GW406731X | 2.72 | GW459057A | 2.01 |

**Supplementary file 1**
