# Supplementary material for: Inhibition of ErbB kinase signalling promotes resolution of neutrophilic inflammation
Source: eLife. 2019 Oct 15;8:e50990. doi: 10.7554/eLife.50990 (PMC6839918; doi:10.7554/eLife.50990)
Supplement: Transparent reporting form [file elife-50990-transrepform.docx]

***eLife’s* transparent reporting form**

We encourage authors to provide detailed information *within their submission* to facilitate the interpretation and replication of experiments. Authors can upload supporting documentation to indicate the use of appropriate reporting guidelines for health-related research (see [EQUATOR Network](http://www.equator-network.org/%20)), life science research (see the [BioSharing Information Resource](https://biosharing.org/)), or the [ARRIVE guidelines](http://www.plosbiology.org/article/info:doi/10.1371/journal.pbio.1000412) for reporting work involving animal research. Where applicable, authors should refer to any relevant reporting standards documents in this form.

If you have any questions, please consult our Journal Policies and/or contact us: [editorial@elifesciences.org](mailto:editorial@elifesciences.org).

**Sample-size estimation**

- You should state whether an appropriate sample size was computed when the study was being designed
- You should state the statistical method of sample size computation and any required assumptions
- If no explicit power analysis was used, you should describe how you decided what sample (replicate) size (number) to use

Please outline where this information can be found within the submission (e.g., sections or figure legends), or explain why this information doesn’t apply to your submission:

No explicit power calculation was used. For human, mouse and zebrafish studies, sample size estimations were based on previous experiments which yield reproducible differences and also what is accepted in the literature for these assays. Initial screening in human cells was performed only once because of availability of compound and the scale of the experiment, but this was validated in 3 further replicates (n=3) with a refined set of compounds. Sample sizes are stated in the figure legends.

**Replicates**

- You should report how often each experiment was performed
- You should include a definition of biological versus technical replication
- The data obtained should be provided and sufficient information should be provided to indicate the number of independent biological and/or technical replicates
- If you encountered any outliers, you should describe how these were handled
- Criteria for exclusion/inclusion of data should be clearly stated
- High-throughput sequence data should be uploaded before submission, with a private link for reviewers provided (these are available from both GEO and ArrayExpress)

Please outline where this information can be found within the submission (e.g., sections or figure legends), or explain why this information doesn’t apply to your submission:

Number of experimental replicates, and ‘n’, are included in each figure legend.

For the human studies, each ‘n’ consisted of an independent human subject and each ‘n’ was conducted on separate days. We considered each ‘n’ as a biological replicate. Within each biological replicate, duplicate technical replicates were performed. We did not screen for outliers.

For mouse studies, each mouse was considered a biological replicate and an ‘n’.

For the zebrafish screen, three larvae were placed in each well containing a separate compound. This was repeated three times on three different days, a score between 0 and 3 was given for each well in each experiment and the mean of these scores determined whether or not a compound matched our criteria for defining a ‘hit’ compound.

For the zebrafish experiments presented in Figure 5, larvae were incubated in a 24-well plate or a 6-well plate with one treatment per well, containing approximately 10-15 larvae per experimental repeat. Total number of larvae (usually 10-15 per experiment, and three independent experiments) are included in figure legends. Each larvae was considered a biological replicate and each icon in graphs represents data from one larvae.

**Statistical reporting**

- Statistical analysis methods should be described and justified
- Raw data should be presented in figures whenever informative to do so (typically when N per group is less than 10)
- For each experiment, you should identify the statistical tests used, exact values of N, definitions of center, methods of multiple test correction, and dispersion and precision measures (e.g., mean, median, SD, SEM, confidence intervals; and, for the major substantive results, a measure of effect size (e.g., Pearson's r, Cohen's d)
- Report exact p-values wherever possible alongside the summary statistics and 95% confidence intervals. These should be reported for all key questions and not only when the p-value is less than 0.05.

Please outline where this information can be found within the submission (e.g., sections or figure legends), or explain why this information doesn’t apply to your submission:

An overview of statistical analysis is given in the Materials and Methods section. Methods for statistical analysis in individual experiments, statistical differences (inc. p value categories) and ‘n’ numbers are reported in the figure legends. Raw data are presented throughout with the exception of the initial round of screening (Figure 1B) which was accumulated from several independent experiments each with their own baseline control values and therefore expressed as fold change, and densitometry for immunoblots (Figure 3F-G) which show ratio of target over loading control.

(For large datasets, or papers with a very large number of statistical tests, you may upload a single table file with tests, Ns, etc., with reference to sections in the manuscript.)

**Group allocation**

- Indicate how samples were allocated into experimental groups (in the case of clinical studies, please specify allocation to treatment method); if randomization was used, please also state if restricted randomization was applied
- Indicate if masking was used during group allocation, data collection and/or data analysis

Please outline where this information can be found within the submission (e.g., sections or figure legends), or explain why this information doesn’t apply to your submission:

Groups were allocated according to treatment conditions or disease (e.g COPD) as indicated in the legends. Mice and zebrafish were randomly allocated into groups. In human neutrophil *in vitro* experiments, neutrophils from each donor were exposed to all treatments indicated in a 96-well plate format.

**Additional data files (“source data”)**

- We encourage you to upload relevant additional data files, such as numerical data that are represented as a graph in a figure, or as a summary table
- Where provided, these should be in the most useful format, and they can be uploaded as “Source data” files linked to a main figure or table
- Include model definition files including the full list of parameters used
- Include code used for data analysis (e.g., R, MatLab)
- Avoid stating that data files are “available upon request”

Please indicate the figures or tables for which source data files have been provided:

Source data have not been provided at this time.
